# Biofabricated 3D in vitro model of fibrosis‐induced abnormal hepatoblast/biliary progenitors' expansion of the developing liver

**DOI:** 10.1002/btm2.10207

**Published:** 2021-06-05

**Authors:** Matthew Brovold, Dale Keller, Mahesh Devarasetty, Anthony Dominijanni, Rohan Shirwaiker, Shay Soker

**Affiliations:** ^1^ Wake Forest Institute for Regenerative Medicine Wake Forest Baptist Medical Center, Medical Center Boulevard Winston‐Salem North Carolina USA; ^2^ Department of Industrial and Systems Engineering North Carolina State University Raleigh North Carolina USA

**Keywords:** 3D organoids, biliary development, fibrosis, HepaRG, hepatic stellate cell

## Abstract

Congenital disorders of the biliary tract are the primary reason for pediatric liver failure and ultimately for pediatric liver transplant needs. Not all causes of these disorders are well understood, but it is known that liver fibrosis occurs in many of those afflicted. The goal of this study is to develop a simple yet robust model that recapitulates physico‐mechanical and cellular aspects of fibrosis mediated via hepatic stellate cells (HSCs) and their effects on biliary progenitor cells. Liver organoids were fabricated by embedding various HSCs, with distinctive abilities to generate mild to severe fibrotic environments, together with undifferentiated liver progenitor cell line, HepaRG, within a collagen I hydrogel. The fibrotic state of each organoid was characterized by examination of extracellular matrix (ECM) remodeling through quantitative image analysis, rheometry, and qPCR. In tandem, the phenotype of the liver progenitor cell and cluster formation was assessed through histology. Activated HSCs (aHSCs) created a more severe fibrotic state, exemplified by a more highly contracted and rigid ECM, as well higher relative expression of *TGF‐β*, *TIMP‐1*, *LOXL2*, and *COL1A2* as compared to immortalized HSCs (LX‐2). Within the more severe fibrotic environment, generated by the aHSCs, higher Notch signaling was associated with an expansion of CK19^+^ cells as well as the formation of larger, more densely populated cell biliary like‐clusters as compared to mild and non‐fibrotic controls. The expansion of CK19^+^ cells, coupled with a severely fibrotic environment, are phenomena found within patients suffering from a variety of congenital liver disorders of the biliary tract. Thus, the model presented here can be utilized as a novel in vitro testing platform to test drugs and identify new targets that could benefit pediatric patients that suffer from the biliary dysgenesis associated with a multitude of congenital liver diseases.

## 1 INTRODUCTION

The principle cause of pediatric liver failure, and thus transplant need, is derived from a small subset of congenital cholangiopathies that can, in some cases, manifest in utero. Physiologically, these disorders result in abnormal cellular development or increased hepatoblast proliferation, resulting in tissue malformations during critical phases of biliary tubulogenesis.^1^, ^2^ These ductal plate malformations (DPMs) cause the bile ducts to become dilated or cystic and affect their ability to maintain normal bile flow through the entire biliary tract resulting in cholestasis.^1^ Major lifelong comorbidities include portal hypertension, cirrhosis, and an increased likelihood of hepatocarcinoma and cholangiocarcinoma.^3^ Examples of congenital liver disease include biliary atresia (BA), congenital hepatic fibrosis (CHF), progressive familial intrahepatic cholestasis (PFIC), Caroli's syndrome (CS), and Alagille syndrome (AGS), which affect 1 in 13,000–70,000.^3^, ^4^ There are few treatment options beyond liver transplantation,^2^, ^5^ but approximately half of all total pediatric transplant need is due to BA and the consequent effects of fibrosis.^2^ A commonality of many biliary tract developmental abnormalities is the prevalence of fibrosis within the liver tissue.^4^ Fibrosis is the abnormal deposition of extracellular matrix (ECM) caused by a combination of ECM overproduction and the failure to effectively proteolyze these overabundant proteins ultimately resulting in reduced organ function, cirrhosis, or organ failure. Fibrosis and its mechanisms have been studied extensively in adults, but data is limited on the effects of fibrosis during liver development and congenital liver disorders.

Fibrosis is driven by specific cellular phenotype, ECM protein synthesis, and ECM remodeling. As healthy liver progresses into a fibrotic state, there is a large increase in ECM protein synthesis such as collagens I, III, IV, fibronectin, and hepatic stellate cells (HSCs) contraction, which collectively can increase tissue stiffness.^6^ The increase in overall stiffness of the fibrotic liver tissue affects function and growth of resident liver cells, and can induce liver progenitor cells to become susceptible to increased Notch signaling.^6^, ^7^, ^8^, ^9^ Animal models of congenital liver diseases are costly, time‐consuming, may not accurately depict human cellular responses, and lack ability to vary the level of fibrosis to study the resulting abnormalities. Human congenital disease models are difficult to create in vitro since they rely on multiple cell types and aberrant regulation of multiple factors that control tissue development. Most in vitro models of human disease employ human cells in 2D (monolayer) culture systems and genetic manipulation of human embryonic stem cells (hESCs) in vitro.^10^, ^11^, ^12^ Gene editing developments in mature human cells and hESCs yielded several new in vitro models; however, most of these target a single cell type and do not replicate the intricate cell–cell interactions between multiple cell types or cell–matrix interactions with ECM proteins during tissue and organ development. In the current study, we describe a 3D coculture system (tissue organoids) composed of human liver progenitor cells and HSCs as a simple and more robust model for use as a drug discovery platform, which replicates aspects of liver fibrosis and abnormal biliary development associated with various liver developmental disorders. To our knowledge, this study is the first in vitro organoid model that compared the effects of differential fibrotic environments, induced by HSCs, on biliary progenitor cells.

## 2 MATERIALS AND METHODS

### 2.1 Cell culture

We isolated human primary fetal HSC according to previous reports^13^, ^14^ and passaged on tissue culture plastic in DMEM 4500 mg/L glucose, 2% (v/v) FBS. Passages 3–10 were used for experiments. The primary HSCs become activated through passaging, acquiring a highly fibrotic phenotype and referred to as activated HSCs (aHSCs).^15^ LX‐2 cells, which are immortalized, human, semi‐aHSCs, provided by Dr. Scott Friedman (Icahn School of Medicine at Mount Sinai, New York, NY), were cultured in 5% CO_2_ at 37°C in DMEM, 4500 mg/L glucose, 2% (v/v) fetal bovine serum (FBS). Passages 6–10 were used during the experiments. HepaRG cells (Biopredic ©), which are immortalized, human, undifferentiated, biopotential hepatoblasts, were cultured according to the manufacturer specifications using the proprietary HepaRG growth media and used up to passage 20.

### 2.2 Organoid construction and culture

Three different organoids were manufactured by the incorporation of either HepaRG, HepaRG+aHSC, or HepaRG+LX‐2 and encapsulated within a collagen I hydrogel. Cells in two‐dimensional culture were dissociated in 0.05% Trypsin–EDTA (0.05%), phenol red (ThermoFisher Scientific ©) and neutralized by the respective maintenance media. Cells were counted via hemocytometer. Cells were dispensed into 15 mL centrifuge tubes, centrifuged at 1200 rpm for 5 min and the supernatant removed. A 1 mL/mg solution of collagen I (rat‐tail) (BD Biosciences ©), PBS (ThermoFisher Scientific ©), dH_2_O, and NaOH was prepared beforehand and kept on ice. This solution was added to the cell pellet, and the cells dispersed by repeated pipetting, resulting in a solution with a cellular concentration of 5.0 × 10^6^/mL. One hundred microliters of solution was dispensed into each well of a PDMS mold (used to contain the solution until full polymerization of the hydrogel is achieved^16^) resulting in 5.0 × 10^5^ cells for each monoculture organoid whereas cocultures consisted of 2.5 × 10^5^ cells of each cell type, totaling 5.0 × 10^5^ cells/organoid. Polymerization was achieved by the neutralization of the acidified collagen I by NaOH and by incubation (37°C) for 25 min within a 6‐well plate. One aliquot of HepaRG growth supplement (Biopredic ©) was added to 500 mL of Williams E Media resulting in a HepaRG growth medium. Three milliliters of HepaRG growth medium was then added to the wells. Organoids were incubated (37°C) and maintained by changing the media every other day. Organoids were cultured for up to 3 weeks.

### 2.3 Contraction assay

Contraction assays were used to measure the HSCs' ability to contract the collagen matrix surrounding the embedded cells (i.e., the smaller the organoid, the higher the degree of contraction). All contraction appeared to take place within the first 48 hours, but out of an abundance of caution, analysis was done after 7 days. An image was taken at day 7, and contraction was calculated by image analysis. The surface area of each organoid was calculated via Adobe Photoshop and ImageJ. The magic wand tool was used to isolate the organoid from the background image. All organoids were copied as layers to a document including a ruler used as a scale bar. Images were converted to an 8‐bit black and white. Organoid diameters were measured in triplicate, and the surface area calculated then were normalized to HepaRG monoculture in terms of percentage of contraction (*N* = 8).

### 2.4 Rheology and elastic modulus calculation

Elastic modulus was determined through generation of a force–displacement curve through compression testing on a TA Instruments HR‐2 Discovery rheometer equipped with a flat, 8 mm, round geometry. Individual organoids were placed onto the center of the rheometer stage and excess liquid removed. The geometry was positioned 2.5 mm above plate without sample contact. The cylindrical samples were compressed at a constant speed (10 μm/s) where force and gap distance measurements were collected every 0.25 s. Sample area was calculated using diameter measurement via image analysis. Stress values were generated by Equation (1). Sample height was calculated from the measured force values contained within the output data set where the first value of increasingly positive values >0.005 N was noted and the corresponding height of the geometry above the plate was recorded as the sample height. Strain values were generated by Equation (2). Stress and strain were then plotted against one another to yield a stress–strain curve consisting of two phases: an initial amorphous phase and a subsequent crystalline phase occurring after a curve elbow. Young's modulus was calculated by finding the slope of the amorphous phase (Supporting Information Figure S1).

(1) $$\sigma =\frac{\text{Force}}{\text{Area}}$$

(2) $$\varepsilon =\frac{\text{Height}-\mathrm{Gap}\;\text{Distance}}{\text{Height}}$$

### 2.5 Microscopy

Images were captured with an Olympus BX63 microscope (Olympus; Center Valley, PA) utilizing the Olympus DP80 camera (Olympus) and a motorized stage. H&E stained slides were imaged utilizing a brightfield light source whereas picrosirius red stained slides were imaged using linearly polarized light and immunofluorescent stained slides were imaged utilizing laser excitation.

### 2.6 CT‐FIRE and CurveAlign analysis

CT‐FIRE and CurveAlign is a program designed by the Laboratory for Optical and Computational Instrumentation at the University of Wisconsin, which uses quantifiable parameters of fiber alignment.^17^ FFPE blocks were cut and stained with the Picrosirius red (PS‐red), then imaged using polarized light microscopy with a linear polarizing filter. Images captured had equal exposure in each group within a statistical test. Organoids were imaged via motorized stage and image tiling. Tiled images were imported into Adobe Photoshop, converted to grayscale, and a universal black level was chosen for background normalization. Three 500 × 500‐pixel regions of interest (ROI) were selected from each organoid totaling 9 ROI per group (*N* = 3). Images were imported into CT‐FIRE, which was used to analyze the collagen fibers, and here used to assess overall ECM remodeling capabilities of the HSC's. Fibers are isolated from images by identifying edges (curvelet transform [CT]) and fiber extraction (FIRE) algorithm and connecting those edges to segment total fibers. These segmented fibers are then analyzed to generate histograms of fiber parameters, such as angle, width, length, and straightness. CurveAlign analysis was performed using the same method. Default settings were used with the exception of the sampling rate, which was changed to 0.06 instead of the default 0.001 to allow for higher levels of sampling. Initial validation testing was carried out on fibrotic human liver tissue. Human liver tissue was obtained under an approved protocol from the Wake Forest University Health Sciences IRB approved on 02/04/20.

### 2.7 Visiopharm image analysis

Visiopharm™ is an image analysis platform allowing for the assessment of IHC stained proteins, which can be used to infer cell morphology, counts, protein expression, and colocalization among other analysis. FFPE sections were stained using standard IFC protocols using primary antibodies from Leica: Novocastra™ liquid mouse monoclonal antibody Cytokeratin‐19 (CK19), Abcam: Anti‐activated Notch‐1 antibody and secondary antibodies from Abcam. Fluorescent images were captured by microscope. Images were imported as uncompressed files into Visiopharm™ software (Broomfield, CO) for analysis and quantification. A script was written to deconvolve each immunofluorescence signal, then isolate the stained CK19^+^ cells, which specifically labels HepaRG cells. After HepaRG cells were segmented, a second script was written to deconvolve the fluorescence signal and quantify the expression or localization of Notch intracellular domain (NICD) positive cells. An additional script was also written to quantify DAPI in order to calculate the total number of cells within the organoid. Each group had three biological replicates, and whole organoids were assessed. These results were imported in Excel as a percentage by dividing the number of positive cells by the number of DAPI stained nuclei.

### 2.8 RNA isolation and qPCR

Organoids were stored in RNALater (Qiagen ©) at −20°C for RNA extraction. RNA was extracted using Fibrous Tissue RNA kit (Qiagen ©) (*N* = 4–6). One to two steel beads were used for each organoid in the TissueLyser (Qiagen ©) for a period of 5 min. The remaining steps of the RNA extraction were performed according to the manufacturer's instructions (Qiagen ©). Equimolar concentrations of RNA were converted to cDNA using the High‐Capacity cDNA Reverse Transcription Kit (Applied Biosystems by ThermoFisher Scientific ©) according to the manufacturer's instructions. qPCR was accomplished by using PowerSYBR Green PCR Master Mix (Applied Biosystems by ThermoFisher Scientific ©) in a 96‐well plate using the QuantStudio 3 Real‐Time PCR System ©. Each condition was performed in triplicate. Cycling conditions included hot activation (50°C for 2 min followed by 95°C for 10 min), amplification (95°C for 15 s, 60°C for 1 min) repeated 40 times, and quantification by SYBR Green fluorescence measurement. Automated thresholding was used to acquire ΔCT of each sample. β2M was selected as the housekeeping gene. qPCR results were calculated by using the Livak method. Sample Ct values were normalized to housekeeping gene (ΔCt) and then normalized to either the control, the 2D counterpart or to LX‐2 depending on the comparison made (ΔΔCt). The resultant ΔΔCt was transformed to fold change by the formula 2̂‐ΔΔCt. To make the larger number more manageable for graphing purposes, the log_2_ was taken. Log values greater than 0.59 are equivalent to a fold change greater than 1.5. Primer sets were selected from the open source Harvard PrimerBank © database and the following were used:

*β2M* (Forward, F) *5′‐GAGGCTATCCAGCGTACTCCA‐3′* and (Reverse, R) *5′‐CGGCAGGCATACTCATCTTTT‐3′*;
*MMP9* (F) *5′‐GGGACGCAGACATCGTCATC‐3′* and (R) *5′‐TCGTCATCGTCGAAATGGGC‐3′*;
*TGFβR1* (F) *5′‐CACAGAGTGGGAACAAAAAGGT‐3′* and (R) *5′‐CCAATGGAACATCGTCGAGCA‐3′*;
*TGFβR2* (F) *5′‐GTAGCTCTGATGAGTGCAATGAC‐3′* and (R) 5*′*‐*CAGATATGGCAACTCCCAGTG‐3′*;
*COL1A2* (F) *5′‐GGCCCTCAAGGTTTCCAAGG‐3′* and (R) *5′‐CACCCTGTGGTCCAACAACTC‐3′*;
*LOXL2* (F) *5′‐AGGACATTCGGATTCGAGCC‐3′* and (R) *5′‐CTTCCTCCGTGAGGCAAAC‐3′*;
*CCL2* (F) *5′‐CCTTCTGTGCCTGCTGCTCATAG‐3′* and (R) *5′‐TCTTCGGAGTTTGGGTTTGCTTGT‐3′*;
*MMP2* (F) *5′‐GGCCCTGTCACTCCTGAGAT‐3′* and (R) *5′‐GGCATCCAGGTTATCGGGGA‐3′*;
*TGFβ1* (F) *5′‐CAATTCCTGGCGATACCTCAG‐3′* and (R) *5′‐GCACAACTCCGGTGACATCAA‐3′*;
*TIMP1* (F) *5′‐ACCACCTTATACCAGCGTTATGA‐3′* and (R) *5′‐GGTGTAGACGAACCGGATGTC‐3′*.

### 2.9 Statistical analysis

Statistical analysis was performed using Matlab, Graphpad and Excel. Means were compared between respective groups. Group means were analyzed by *t*‐test, with a confidence interval of 95% leaving *p* < 0.05 as statistically significant. Error bars on graphs are shown as SD. Boxplots generated by Matlab display the IQR of all data points and visually expresses significance (*p* < 0.05) by non‐overlapping notches at the mean across the *x*‐axis. Angle variance itself is a circular statistic that expresses overall alignment of all fibers analyzed.

## 3 RESULTS

### 3.1 aHSCs induce contraction and stiffening of 3D organoids

**FIGURE 1:**
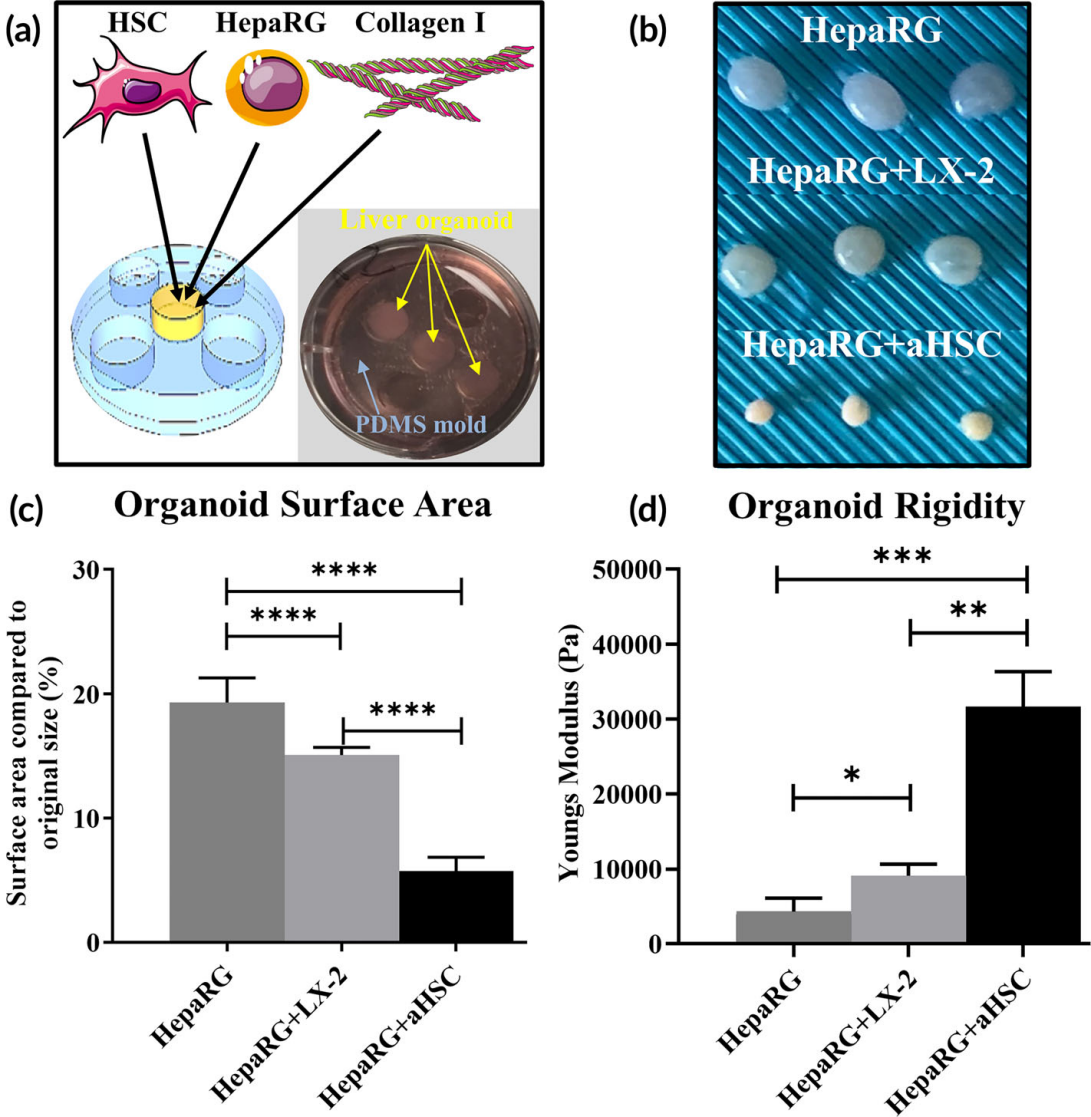
Fabrication and contraction of liver organoids. (a) Schematic of organoid construction. Organoids were fabricated by mixing HepaRG cells, collagen I, and one of two types of HSC and dispensing into a PDMS mold, as described in the Methods (shown are mold design (left) and organoids in culture (right)). (b) A macroscopic view of the different liver organoid types as indicated. (c) Surface area analysis of the different organoid types after 1 week in culture showing a significant contraction for HepaRG+aHSC organoids compared with HepaRG and HepaRG+LX‐2 organoids (****p < 0.0001, *N* = 8). (d) Young's modulus of the different liver organoids was calculated using rheological analysis, as described in the Methods. aHSC and HepaRG+aHSC organoids show significantly higher stiffness than other organoid types (***p ≤ 0.001, **p ≤ 0.01, *p ≤ 0.05, *N* = 3)

HSC activation is a major contributor to liver fibrosis in cirrhosis and other liver pathologies.^18^, ^19^, ^20^, ^21^ To create a model for liver fibrosis, we evaluated two different types of HSCs in 3D liver organoids representing intermediate and high activation levels (Supporting Information Figure S2)—LX‐2, an immortalized HSC line of HSC activation (intermediate), and primary HSCs (high) that were activated by serial passaging in tissue culture plastic plates (aHSC) (Figure 1a). HSCs were encapsulated in a collagen I hydrogel, placed in a PDMS mold to allow for self‐assembly and then cultured for up to 3 weeks (Figure 1). To simulate liver development in the 3D organoids, we used an immortalized cell line, HepaRG, which mimics hepatoblasts by developing into hepatocytes and cholangiocytes^22^ and can form primitive duct‐like structures in 3D cultures (Supporting Information Figure S4). We created coculture organoids composed of HepaRG cells with LX‐2 or aHSC cells (2.5 × 10^5^ cells of each cell type/organoid) (Figure 1b). Macroscopic observations showed a similar pattern as observed for the single cell type organoids (Figure 1b), with moderate contraction for organoids composed of HepaRG cells alone, and significantly higher contraction when LX‐2 or aHSC cells were included in the organoids (Figure 1c). As compared to the initial surface area, calculated immediately after dispensing of the cell: collagen solution into the PDMS mold, HepaRG monoculture had the largest surface as compared to both cocultures containing HSCs (*p* = 0.0001). HepaRG organoids had an average 19.3% of the initial surface area, HepaRG+LX‐2 had an average 15.1% of the initial surface area, and HepaRG+aHSC organoids contracted significantly more to an average of 5.7% compared to the initial surface area.

Increased tissue stiffness, a hallmark of a fibrotic liver, is potentially due to a combination of crosslinking of collagen fibers and contraction.^23^, ^24^, ^25^ In a previous study we found a monoculture of aHSC cells created a stiffer environment than the monoculture of LX‐2 cells.^26^ Here we assessed coculture organoid stiffness by rheometric analysis (Figure 1d). As expected, we observed a significant increase in tissue stiffness in organoids that contained LX‐2 or aHSC cells in coculture compared with organoids containing HepaRG cells alone. HepaRG+aHSC organoids with a mean stiffness of 3.1 × 10^4^ Pa were significantly stiffer than HepaRG+LX‐2 organoids (*p* ≤ 0.01), which may be due to the higher overall contraction of the HepaRG+aHSC organoids.

### 3.2 HSCs actively remodel collagen I in the liver organoids

**FIGURE 2:**
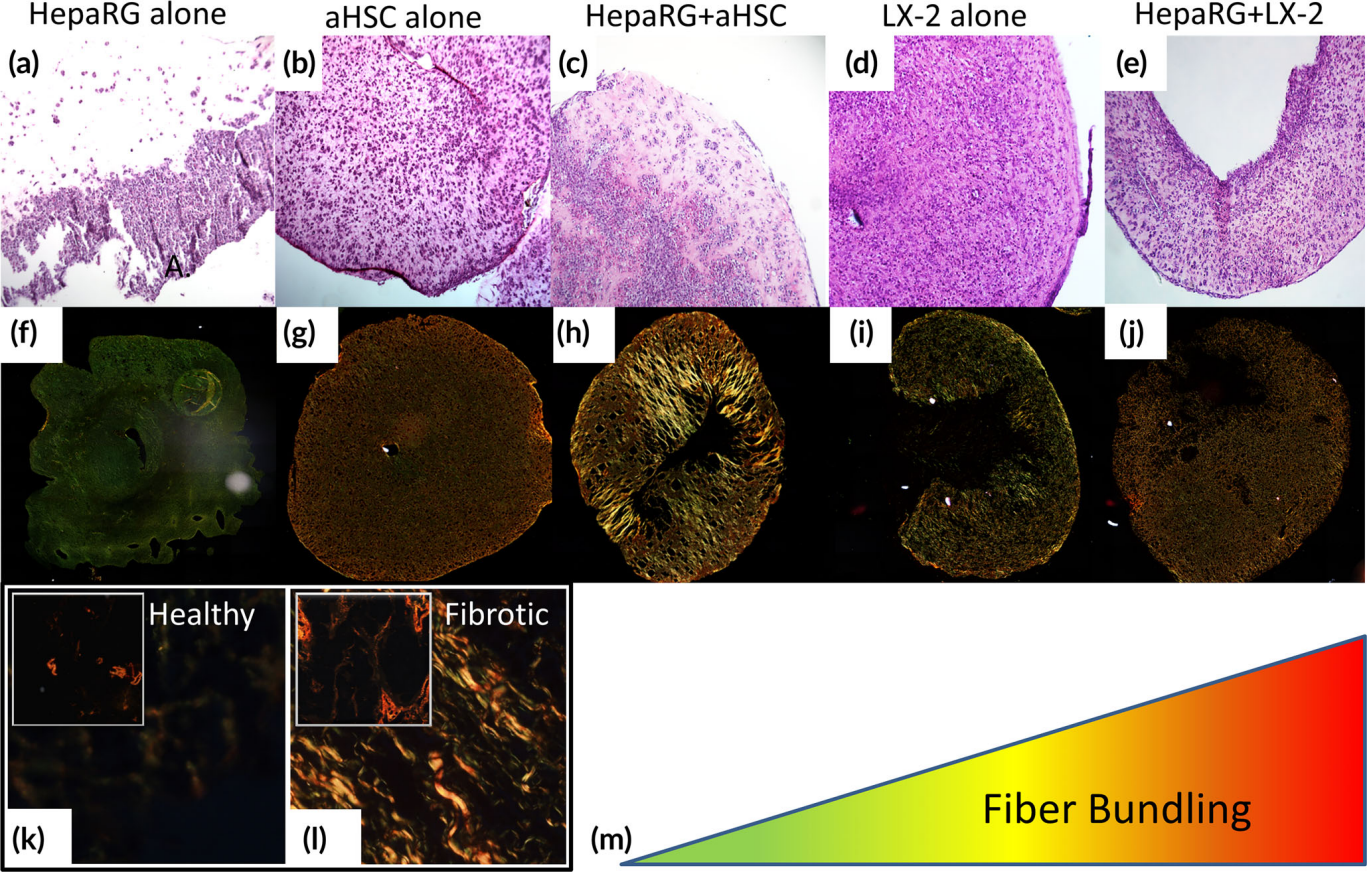
Histological analysis of liver organoids. Different types of liver organoids as indicated, were cultured for 1 week and processed for H&E (a–e) and PS Red (f–j) staining, as described in the Methods. H&E staining show different cellular organization within the organoids (~200× magnification images processed to show whole organoid regardless of actual size). PS Red staining of collagen fibers created colors ranging from green ➔ yellow ➔ orange ➔ bright red that indicate increasing fiber bundling, as depicted in the diagram (m). Samples of healthy and fibrotic human liver tissues (see Methods for statement), as indicated, were processed for PS Red staining for comparison with the liver organoids (k and l). The aHSC and HepaRG+aHSC organoids show higher orange and red staining compared with the other organoid types, and similar to fibrotic human liver tissue (****p ≤ 0.0001, *p ≤ 0.05, *N* = 3)

ECM remodeling is highly regulated and generally associated with development, wound healing, or tissue regeneration. However, when ECM remodeling becomes unregulated leads to excessive collagen production and collagen hyper‐bundling, via crosslinking, yielding a denser and stiffer ECM subsequently resulting as tissue fibrosis.^27^ The results described above indicated that collagen within the organoids underwent conformational changes in the presence of LX‐2 and aHSC cells. To determine if these changes are associated with collagen remodeling through crosslinking and bundling, we carried out histological staining and image analysis of collagen fibers. Initial H&E staining shows a difference in the organoid's cellular organization. Cells within HepaRG organoids localized to one side of the organoid, conversely cells within organoids containing LX‐2 and aHSC are evenly distributed (Figure 2). Coculture organoids had similar uniform cell distribution; in addition, these organoids showed formation of cellular structures at the center of the organoids, especially those of HepaRG+aHSC organoids (Figure 2c).

PS‐Red staining allows for the visualization of the overall collagen fiber organization and also has the ability to distinguish between highly bundled (thick, red to orange color) and unbundled (reticular, dark to light green color) collagen fibers via polarized light microscopy. PS‐Red imaging indicated that organoids containing HepaRG cells alone are mainly green or unbundled collagen fibers. LX‐2 and aHSC organoids, alone or cocultured with HepaRG, are mostly orange to bright red (Figure 2f–j). Specifically, aHSC organoids are orange while the HepaRG+aHSC organoids are bright red, especially in the center of the organoids. LX‐2 organoids are shown as green to orange in color, with some brighter lines throughout. Healthy and fibrotic liver samples showed a similar trend. Bundled collagen is observed only around the hepatic portal structures and rarely in the parenchyma (Figure 2k). In contrast, fibrotic liver samples showed thick and bundled collagen throughout the parenchyma with bridging fibrosis connecting the septa and the portal triad, including the biliary tract and the vasculature (Figure 2l).

### 3.3 Quantitative analysis of collagen I fibers in the liver organoids

**FIGURE 3:**
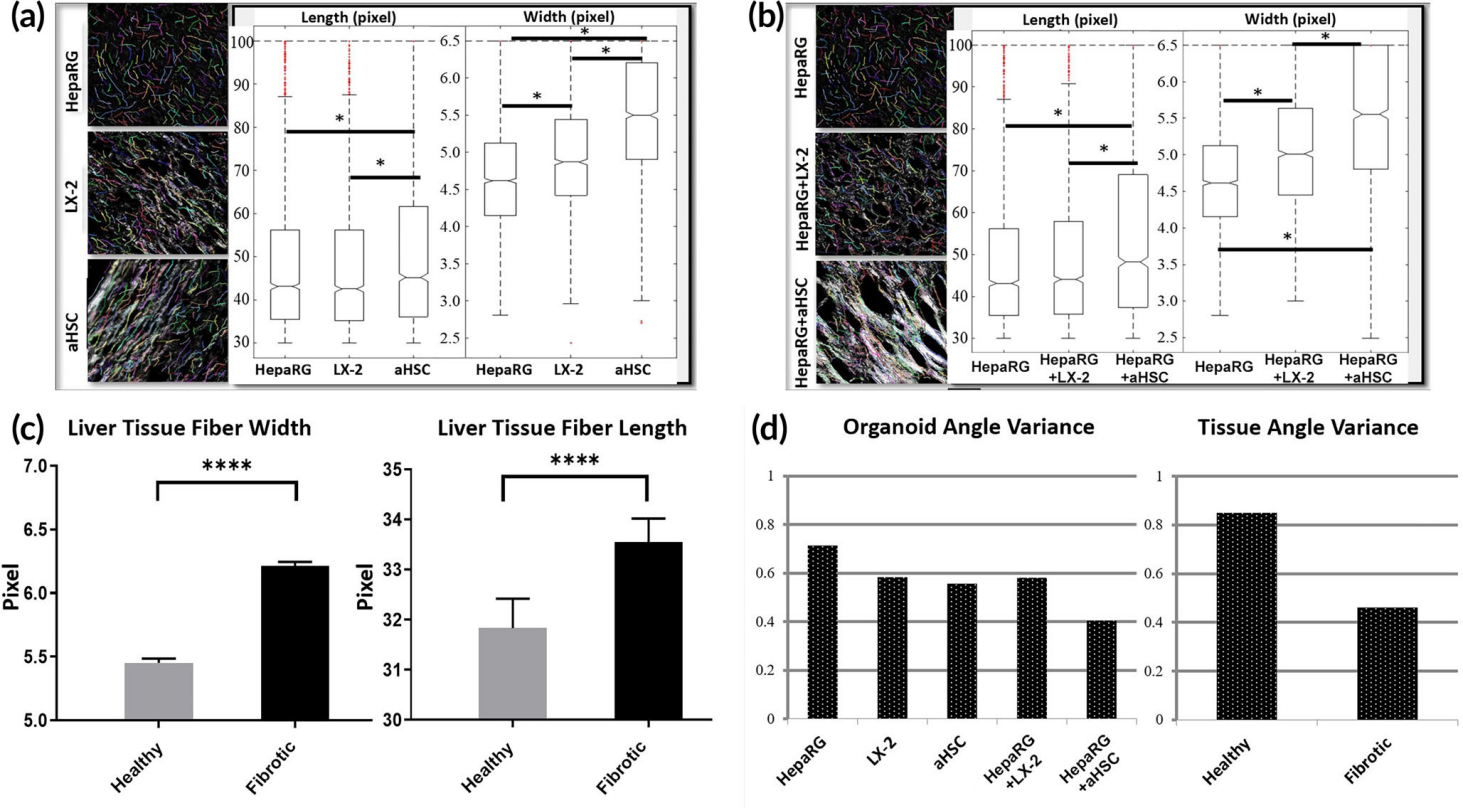
Analysis of fiber properties in the liver organoids. The PS Red images from Figure 2 of monocultures (a) and cocultures (b) organoids and fibrotic and healthy and fibrotic human liver tissues (c) were analyzed with the CT‐FIRE™ program, and fiber length and width (pixels) were calculated. (d) Angle variance of fibers in organoids and human liver tissues depicts overall alignment of all fibers, where a statistic of 0 indicates all fibers are completely aligned and 1 indicated random organization. Organoids containing aHSC alone and HepaRG+aHSC cocultures have longer and wider fibers, with lower angle variance, compared with the other organoids (*N* = 3). Similarly, fibrotic liver tissue has longer and wider fibers, with lower angle variance, compared with healthy liver tissue

To characterize collagen I remodeling in the liver organoids, we analyzed PS‐Red images by quantifying the collagen fiber properties using CT‐FIRE. Organoids containing only HepaRG cells had equal fiber length compared to LX‐2 organoids, but LX‐2 organoids had significantly thicker fibers. Organoids containing aHSC cells alone had significantly longer and thicker fibers than HepaRG and LX‐2 organoids (Figure 3a). HepaRG+LX‐2 organoids did not show longer fibers than HepaRG organoids, but HepaRG+LX‐2 organoid fibers were thicker. HepaRG+aHSC organoids showed fibers significantly longer and thicker than within HepaRG+LX‐2 organoids (Figure 3b). In a previous study we showed that a monoculture of aHSC cells created longer and thicker fibers than the monoculture of LX‐2 cells.^26^ Similar analysis of collagen fibers were carried out in healthy and cirrhotic liver specimens. In comparison, cirrhotic liver tissue contained fibers that were thicker, wider, and more highly aligned (Figure 3c). The distribution of collagen fiber angles was observed, the circular statistic was calculated presented on a scale from 0 to 1, where 0 indicates all fibers being fully aligned and 1 indicates completely unaligned fibers (Figure 3d). HepaRG organoid fiber alignment appears random, confirming the similar trend of the lower fiber length and width values. LX‐2 or aHSC organoids showed higher alignment than HepaRG organoids. HepaRG+LX‐2 organoids had similar alignment as the LX‐2 organoids, whereas HepaRG+aHSC organoids showed the highest fiber alignment. Similar trends in fiber alignment were observed when comparing healthy and fibrotic liver tissue specimens. Fibrotic liver tissue had higher fiber alignment than healthy liver tissue.

### 3.4 Analysis of fibrosis‐related pathways in HSCs

**FIGURE 4:**
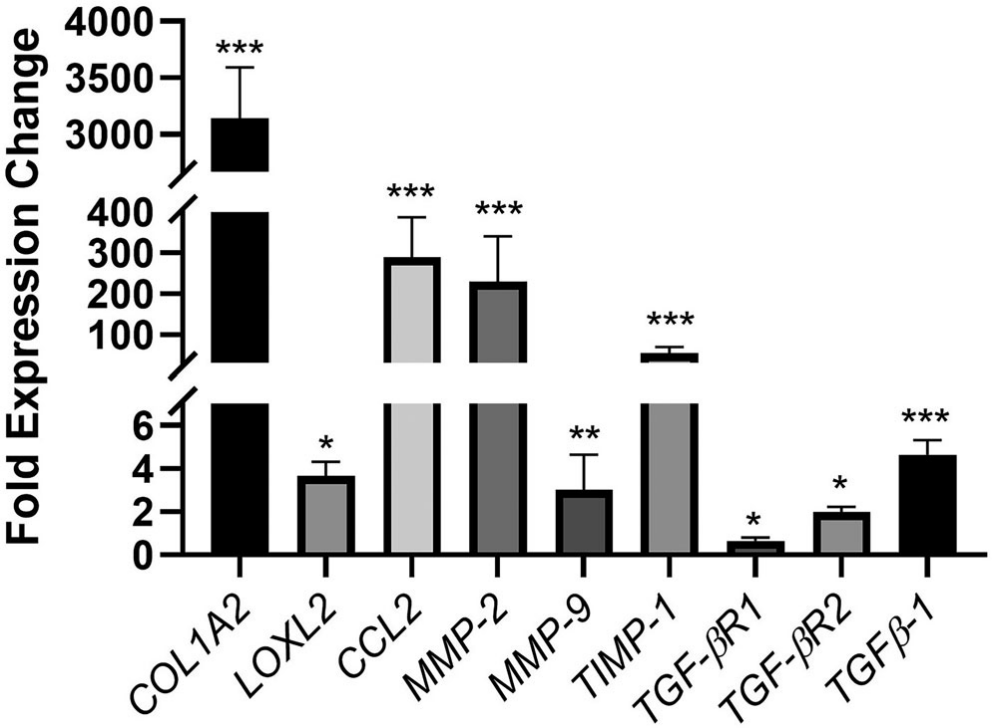
Expression of fibrosis genes in liver organoids containing aHSC and LX‐2 cells. Expression of genes associated with fibrosis in monoculture organoids of aHSC and LX‐2 cells was analyzed using qPCR, as described in the Methods. The results show fold‐expression changes of genes in aHSC organoids compared with LX‐2 organoids. Organoids containing aHSC cells showed higher expression of *COL1A2*, *LOXL2*, *MMP2* and *MMP9* and *TGF‐β1* suggesting active ECM remodeling and an elevated fibrotic state in these organoids compared with LX‐2 containing organoids (***p ≤ 0.001, **p ≤ 0.01, *p ≤ 0.05, *N* = 4–6)

We analyzed fibrosis‐related genes *COL1A2*, *TIMP1*, *TGF‐β1*, *LOXL2*, *MMP2*, *CCL2*, *TGF‐βR1*, *TGF‐βR2*, and *MMP9* (Figure 4) to characterize differences in the organoids. Comparing the relative expression levels in aHSC organoids to those in LX‐2 organoids revealed that aHSC organoids have a higher expression of every gene from the list except for *TGF‐βR1*. Importantly, the aHSC organoids had higher *LOXL2* expression, responsible for the crosslinking/bundling of collagen, corroborated by the fiber analysis results above. The increased expression of *COL1A2*, *MMP2* and *MMP9* in aHSC organoids is indicative of ECM remodeling and an elevated fibrotic state. Additionally, aHSC organoids showed higher *TIMP1 expression*, an MMP inhibitor, which prevents the breakdown of the ECM, another indicator of increasing fibrosis. *TGF‐β1* expression is also of interest as it is an important regulator of biliary tract development and increased expression may have downstream effects on the HepaRG cells.

### 3.5 The effects of HSC‐mediated ECM organization on biliary progenitor cell expansion

**FIGURE 5:**
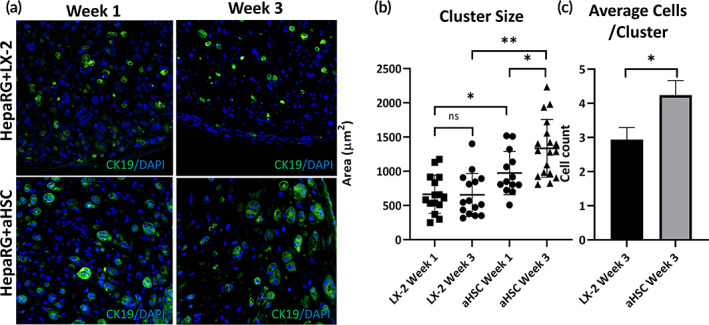
Biliary‐like structure formation in liver organoids. As indicated, organoids were harvested for analysis after 1 and 3 weeks in culture. (a) HepaRG+LX‐2 and HepaRG+aHSC organoids cultured for 1 and 3 weeks, as indicated, were processed for immunohistochemical analysis using anti‐CK19 antibodies and nuclear staining with DAPI. (b and c) The average size (area) of CK19^+^ cell clusters (b) and the average number of CK19^+^ cells per cluster (c) after 3 weeks in culture was calculated from (a). There is significant increase in the cluster size and the number of CK19^+^ cells/cluster in aHSC coculture organoids compared to LX‐2 coculture organoids (**p ≤ 0.01, *p ≤ 0.05, *N* = 3)

Our results exemplify differences in both physical and structural properties between organoids containing aHSC and LX‐2 cells. Specifically, microenvironments with differing rigidity and collagen fiber organization that could impact the growth and differentiation of the HepaRG progenitors. To test this hypothesis, we analyzed HepaRG+aHSC and HepaRG+LX‐2 organoids for the number and sizes of ductular‐like structures (clusters) positive for CK19, a marker of biliary cells (Figure 5a). CK19^+^ cell clusters in the HepaRG+aHSC organoids progressively grew in size (surface area) between week 1 and 3, and were larger than those in surface area in weeks 1 and 3 as compared to HepaRG+LX‐2 organoid (Figure 5b). Furthermore, the average number of cells per cluster in the HepaRG+aHSC organoid was significantly higher than HepaRG+LX‐2 organoids (Figure 5c). Additionally, we compared HepaRG+aHSC organoids to HepaRG organoids, and observed larger cluster size in HepaRG+aHSC organoids (Supporting Information Figure S5).

### 3.6 Effect of differential fibrotic environment on pathways associated with biliary differentiation

**FIGURE 6:**
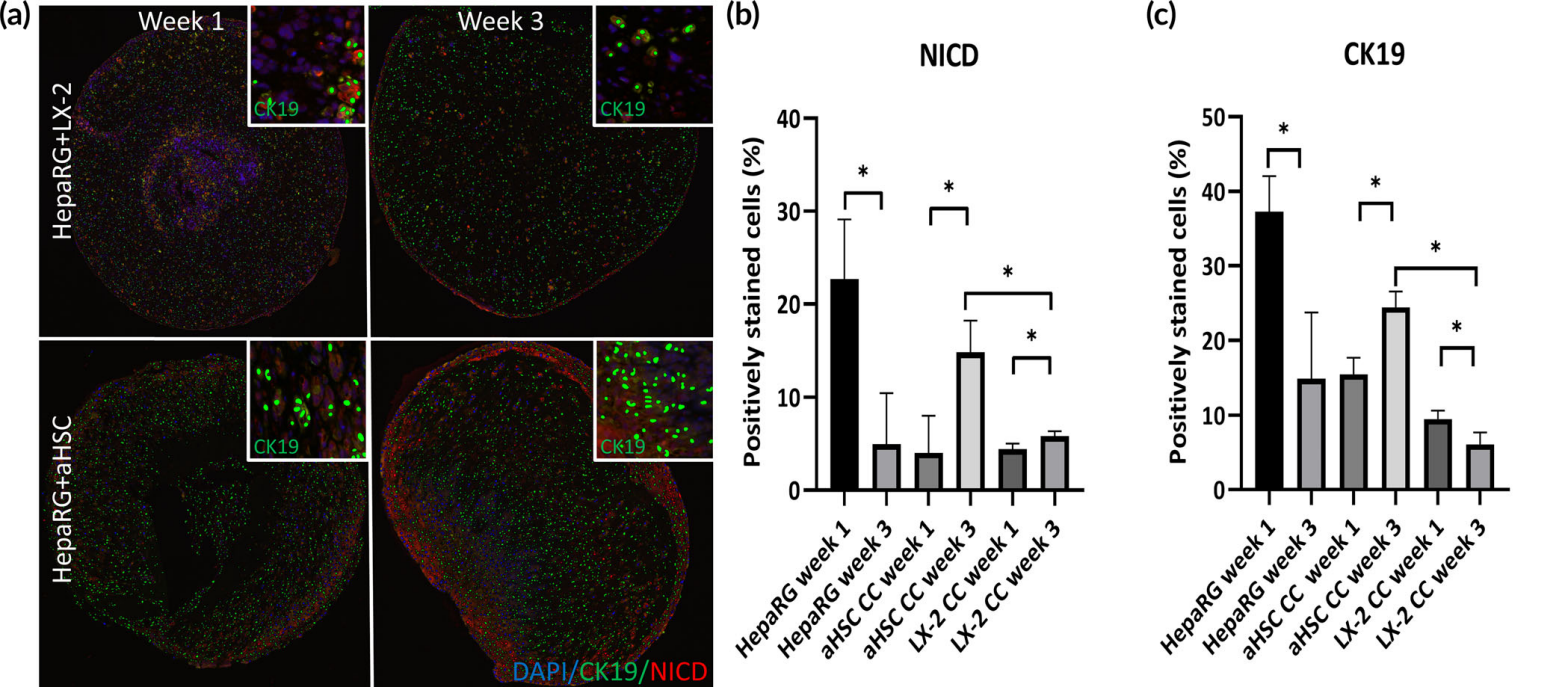
Visiopharm analysis of CK19 and Notch positive cells. (a) Organoids from each respective group were stained for CK19, NICD and DAPI at weeks 1 and 3. The resulting fluorescent with the Visiopharm generated overlays of positive cells for each marker. These images were quantified by Visopharm and analyzed, as described in Methods. Insets are representative images of CK19^+^ cells identified and counted by the Visopharm‐generated overlays. (b) Notch intracellular domain (NICD)‐positive cells were divided by total number of DAPI stained nuclei to calculate the percentage of NICD positive cells. (c) CK19^+^ cells were divided by total number of DAPI stained nuclei to calculate the percentage of CK19^+^. There is a rise in Notch activation and CK19^+^ cells in HepaRG+aHSC coculture organoids between weeks 1 and 3 compared with steady numbers in HepaRG+LX‐2 coculture organoids and a significant decrease in HepaRG monoculture organoids (*p ≤ 0.05, *N* = 3)

To assess the differences in HepaRG cell differentiation, we measured Notch activation and CK19 expression in HepaRG+aHSC and HepaRG+LX‐2 organoids (Figure 6). Notch signaling is important to biliary development generally committing hepatoblasts to a biliary lineage, which could influence the number of CK19^+^ cells in the organoids. Accordingly, we hypothesized that a fibrotic environment would induce Notch signaling and activation and thus contain higher numbers of CK19^+^ cells. Notch activation was assessed by immunostaining for NICD and analyzed using Visiopharm™ software. NICD^+^ cell numbers significantly decreased from week 1 to 3 in HepaRG alone, but increased in HepaRG+aHSC organoids (Figure 6a,b). In contrast, the percentage of NICD^+^ cells remained largely unchanged in HepaRG+LX‐2 organoids. To determine the subsequent effect of Notch signaling in HSCs on hepatoblast expansion, we stained sections for CK19, a marker for hepatoblasts and biliary cells, and analyzed them using Visiopharm™ software. Analysis shows the percentage of CK19^+^ cells decreased from week 1 to 3 in organoids containing HepaRG alone or HepaRG+LX‐2 organoids (Figure 6a,c). In contrast, and similar to Notch activation results, the percentage of CK19^+^ cells increased from week 1 to 3 in HepaRG+aHSC organoids, and the overall percentage of CK19^+^cells in HepaRG+aHSC organoids was significantly higher in these organoids (Figure 6c).

## 4 DISCUSSION

Most models of liver fibrosis have been created to simulate conditions found within adults.^28^, ^29^ Less emphasis is placed on liver fibrosis during fetal development resulting in a lack of reliable models that simulate aspects congenital liver diseases. In this study, we used a liver progenitor cell line, HepaRG, to simulate the major cell type of the developing liver within the organoids and aHSCs to simulate the physical and chemical cues found in a fibrotic environment. HepaRGs are a hepatoblast‐like cell, which differentiates into hepatocyte‐like and cholangiocyte‐like cells,^30^ are used to monitor drug metabolism^31^ and have been shown to form putative biliary duct‐like structures, hepatocyte‐like cells inside a 3D substrate.^32^, ^33^ HepaRG's have been used in models to simulate toxin‐induced BA^34^ however, none in conjunction with activated HSCs, which influence biliary development and liver disease.^18^ The aHSCs participate in the regulation of hepatoblast expansion and differentiation during development becoming apparent during the remodeling stage of the biliary tract. These cells are also the key cellular component of liver fibrosis. In our study both HSC types LX‐2 and aHSC are activated based on α‐SMA expression which is a well‐defined marker for activation.^26^, ^35^, ^36^ As HSCs are normally found in quiescent state in vivo and express low levels of α‐SMA. However, culture in plastic tissue dishes for as little as 12 hours is sufficient to activate the cells. As such, comparison of α‐SMA expression in any cultured HSC is difficult. However, if compared to the expression of a normalizing housekeeping gene, LX‐2 at a minimum, should be considered semi‐activated (Supporting Information Figure S2). However, LX‐2 cells can be further activated by supplementation of exogenous TGF‐β. As HSCs are required for development and are activated at some stage of development, they were included within the constructs. The aHSCs on the other hand represent a highly fibrotic more indicative of what may be found in later stages of fibrosis or in cases of biliary atresia. Most other congenital liver diseases models rely mainly on small animal models and gene manipulations^37^, ^38^ or hepatotoxin treatment.^39^, ^40^, ^41^ For example, the Gunn rat model of inherited bilirubin‐UGT deficiency such as the Crigler‐Najjar syndrome and the inv mouse (partial deletion of the inversin gene) model of biliary atresia (BA)^42^ were helpful in hepatic and biliary diseases studies, respectively. The Jag1dDSL/+ Notch2del1/+ mouse is a model for Alagille syndrome.^43^ These models are not optimal for human‐specific disease studies due to differences in liver fetal development between species. Cocultured together in a 3D environment we endeavor to more simply and robustly replicate aspects of fetal liver fibrosis.

Identifying physico‐mechanical ECM properties, including stiffness and collagen fiber organization, are important for establishing quantitative measures within models that depict varied levels of fibrosis‐like state. We recently published two papers using similar techniques for quantification and analysis of collagen fibers. The first was a 3D organoid colorectal cancer model that included the submucosal tissue with primary colonic SMCs and collagen I and found collagen organization was similar to the colonic ECM in vivo.^16^ The second was a 3D model of liver fibrosis, which effectively replicated aspects of liver fibrosis of different severities when comparing monocultures of aHSC and LX‐2 utilizing similar metrics found within this study.^26^ As fibrosis increases in severity, we generally see an increase of tissue stiffness, collagen production, and TIMP1 and LOXL2 expression, which was found in this study and more extensively illustrated in our previous study where we successfully created two different fibrotic environments generated by different HSC types, one activated by in vitro sub‐culture (aHSC) and the other immortalized (LX‐2); one created a higher stiffness than the other, along with higher fibrosis‐related gene expression levels of *COL1A2*, *TIMP1*, and *LOXL2*.^26^ Additionally, our aHSC constructs more extensively remodeled the ECM, can be found in more severe cases of congenital liver disorders. These results indicate that the presence of aHSCs induced higher contraction of the organoids, increased organoid stiffness, the formation of more highly bundled, longer, and thicker fibers, all of which are often associated with the abnormal anatomy of fibrotic liver tissues.^44^, ^45^

Capitalizing on these differences in fibrotic environments we have used liver progenitor cells (HepaRG), HSCs and a highly abundant liver ECM protein, collagen I in order to create 3D liver organoids. The undifferentiated HepaRG cells better resemble the developing liver compared with the popular HepG2 hepatocyte‐like cells.^46^ Utilizing collagen I to fabricate the liver organoids allows for self‐aggregation of cells and remodeling of the ECM microenvironment within the organoids without the presence of growth factors that may influence HepaRG cell differentiation as with Matrigel™. Recent studies tested various environmental stiffness and cellular combinations on HepaRG differentiation, but used skin fibroblasts,^47^ or the effects of individual environmental toxins on luminal formation.^33^ Neither of these studies utilized activated HSCs that contribute to both cellular signaling and physico‐mechanical ECM properties. In contrast, the coculture of undifferentiated HepaRG cells with the two types of HSCs described above, each capable of generating distinct fibrotic environments, makes our model more relevant for the use in drug studies of fibrosis‐related biliary abnormalities. In a previous study we found that the introduction of anti‐fibrotic (Alk‐5 inhibitor) and pro‐fibrotic (Methotrexate) drugs had significant effect on ECM remodeling, gene expression pathways associated with fibrosis and, in the case for the moderately fibrotic LX‐2, reduction of overall tissue stiffness within the organoids.^26^ Additionally, as these organoids represent several aspects of the fibrotic environment, specifically ECM remodeling and synthesis of soluble factors (TGF‐β) associated with both fibrosis and development (Supporting Information Figure S3), addition of ALK5i effectively reduced TGF‐β gene expression.^26^

During embryonic development hepatoblasts invade the liver diverticulum and begin to proliferate, differentiate into biliary cells and hepatocytes, as well as begin the formation of the bile ducts orchestrated from cues within the fetal mesenchyme.^48^ This high concentration of hepatoblasts diminishes over time as the liver matures due to differentiation, which can be tracked by the reduction of CK19 positive cells (a marker for hepatoblasts and biliary cells). Previously we have shown that albumin producing cell clusters are present next to ck19 positive clusters within liver organoids.^49^ HSCs have an integral role in the normal development of the liver wherein they produce factors that direct development^50^, ^51^; without them, livers develop abnormally and having severely diminished hematopoiesis.^52^, ^53^ As HSCs are the main driver of ECM remodeling^54^ and liver fibrosis, they may also have a role in abnormal liver development as fibrotic HSCs are found in high abundance in cases of biliary atresia (BA) and other cholangiopathies.^55^ HSC and HepaRG coculturing lead to the formation of premature ductular structures similar to those observed with hFLPCs.^49^, ^56^, ^57^ Over time, CK19^+^ structures in the HepaRG+aHSC organoids became larger and had higher number of cells per cluster, which was not found in the HepaRG+LX‐2 organoids or in HepaRG alone cultures. In parallel, we also found an increase in the percentage CK19^+^ cells in the more fibrotic organoid created by the aHSCs. This coincides with a higher level of Notch signaling within the organoid as well. This increased Notch signaling can lead to amplified hepatoblast proliferation, resulting in ductal plate malformations, which has been observed in some cases of BA.^58^ A hallmark of BA pathology is portal‐based fibrosis and the presence of fibrous expansion of the portal tracks with advanced stages of fibrosis.^59^ Accordingly, we propose that the differences in cluster size, expansion of CK19^+^ cells observed between LX‐2 and aHSC containing organoids are in part a result of the more highly fibrotic environment created by the aHSCs in the liver organoids.

Collectively, these results suggest that higher levels of ECM remodeling, collagen bundling, and HSC‐secreted paracrine factors, observed in the HepaRG+aHSC organoids, may have directed the HepaRG cells toward progressive expansion of CK19^+^ immature cells and formation larger clusters induced by higher levels of Notch signaling.

## 5 CONCLUSIONS

Utilizing the 3D liver organoid model, we demonstrated that including aHSC in the organoids resulted in a much stiffer environment with more elongated, highly bundled, and aligned collagen fibers as compared to organoids containing the less activated LX‐2 cells. Organoids containing aHSCs have higher relative *COL1A2*, *LOXL2*, and *TGF‐β* expression, resulting in a fibrosis‐associated phenotype. Either directly resulting from the fibrotic environment or indirectly, organoids containing the more highly activated aHSC cells showed a higher percentage of CK19^+^ cells overall as well as larger clusters of biliary like structures as compared to LX‐2. These results suggest that more fibrotic environments may impact the proliferation of progenitor cells and subsequent development of the biliary tract. This may provide insight into the mechanism of aberrant proliferation of biliary cells in cases of BA. The model system can be easily manufactured and used as a high‐throughput assay, allowing for multiple simultaneous studies of molecular pathways or for pharmaceutical application. The administration of other growth factors and cells such as Kupffer immune cells could further improve the model by simulating the important role of liver inflammation in cirrhosis.^60^ In the future, this organoid system can be used for pharmacological targeting of these pathways to reinstate normal liver development as a treatment for congenital liver disorders.

## AUTHOR CONTRIBUTIONS

**Matthew Brovold:** Conceptualization; data curation; formal analysis; writing‐original draft; writing‐review and editing. **Dale Keller:** Data curation. **Anthony Dominijanni:** Data curation. **Mahesh Devarasetty:** Methodology. **Rohan Shriwaiker:** Writing‐review and editing. **Shay‐ Soker:** Conceptualization; funding acquisition; project administration; supervision; writing‐review and editing.

## CONFLICT OF INTEREST

The author declares that there is no conflict of interest that could be perceived as prejudicing the impartiality of the research reported.

6

### PEER REVIEW

The peer review history for this article is available at https://publons.com/publon/10.1002/btm2.10207.

## Supporting information

**Figure S1** Slope defining Young's modulus. Youngs modulus was calculated by finding the slope of the amorphous phase of the stress strain curve.

**Figure S2** Relative gene expression of activation markers in hepatic stellate cells. A and B, α‐SMA (A) and glial fibrillary acidic protein (GFAP) (B) expression in LX‐2 and aHSC cells. The expression levels were determined by the Livak method, by normalization to the housekeeping gene β2M. C, Collagen IA2 (COL1A2) expression in aHSC cells compared (normalized) with LX‐2 cells.

**Figure S3** IFC staining of TGF‐B in fibrotic organoids. Immune‐fluorescence staining of TGF‐β in organoids reveals higher TGF‐β expression in HepaRG+aHSC organoids compared with HepaRG+ LX‐2 organoids. Additionally, TGF‐β distribution was observed throughout the HepaRG+aHSC organoid, whereas in HepaRG+LX‐2 organoids it is largely located along the periphery.

**Figure S4** Primitive ductular structures (clusters) generated by HepaRG. A, HepaRG form primitive ductular structures when cultured in 3D constructs. Fully mature biliary ducts express F‐actin in the lumen only and β‐catenin in between. These appear to be immature ducts. B, Cell clusters and lumen‐containing structures observed within the organoids.

**Figure S5** Image analysis of primitive biliary structures in liver organoids. HepaRG and HepaRG+aHSC organoids, as indicated, were harvested for analysis after 1 week in culture. A Matlab script was generated to identify CK19+ duct‐like structure within the image. Regions of interest (ROI's) were thresholded for size, removing large and small outliers. Results show the mean and variance of the number of CK19+ duct‐like structures in HepaRG and HepaRG+aHSC organoids. Organoids containing HepaRG+aHSC cocultures showed higher numbers of CK19+ cell clusters compared with HepaRG alone organoids.
