# Supplementary figures and images for: Biofabricated 3D in vitro model of fibrosis‐induced abnormal hepatoblast/biliary progenitors' expansion of the developing liver
Source: Bioeng Transl Med. 2021 Jun 5;6(3):e10207. doi: 10.1002/btm2.10207 (PMC8459590; doi:10.1002/btm2.10207)

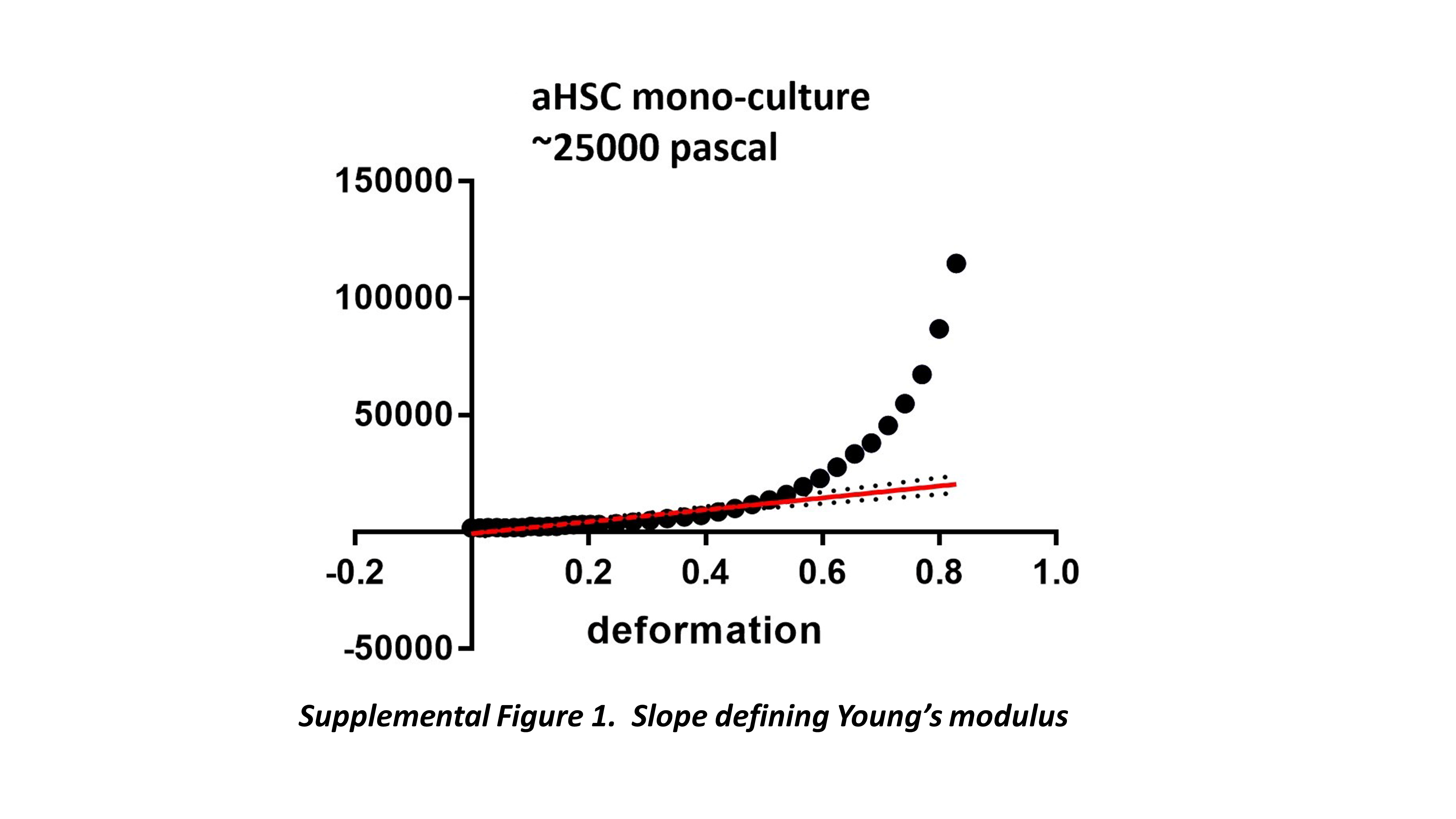

Supplement: Supplementary file 1 — Figure S1 Slope defining Young's modulus. Youngs modulus was calculated by finding the slope of the amorphous phase of the stress strain curve. [file BTM2-6-e10207-s005.TIF]

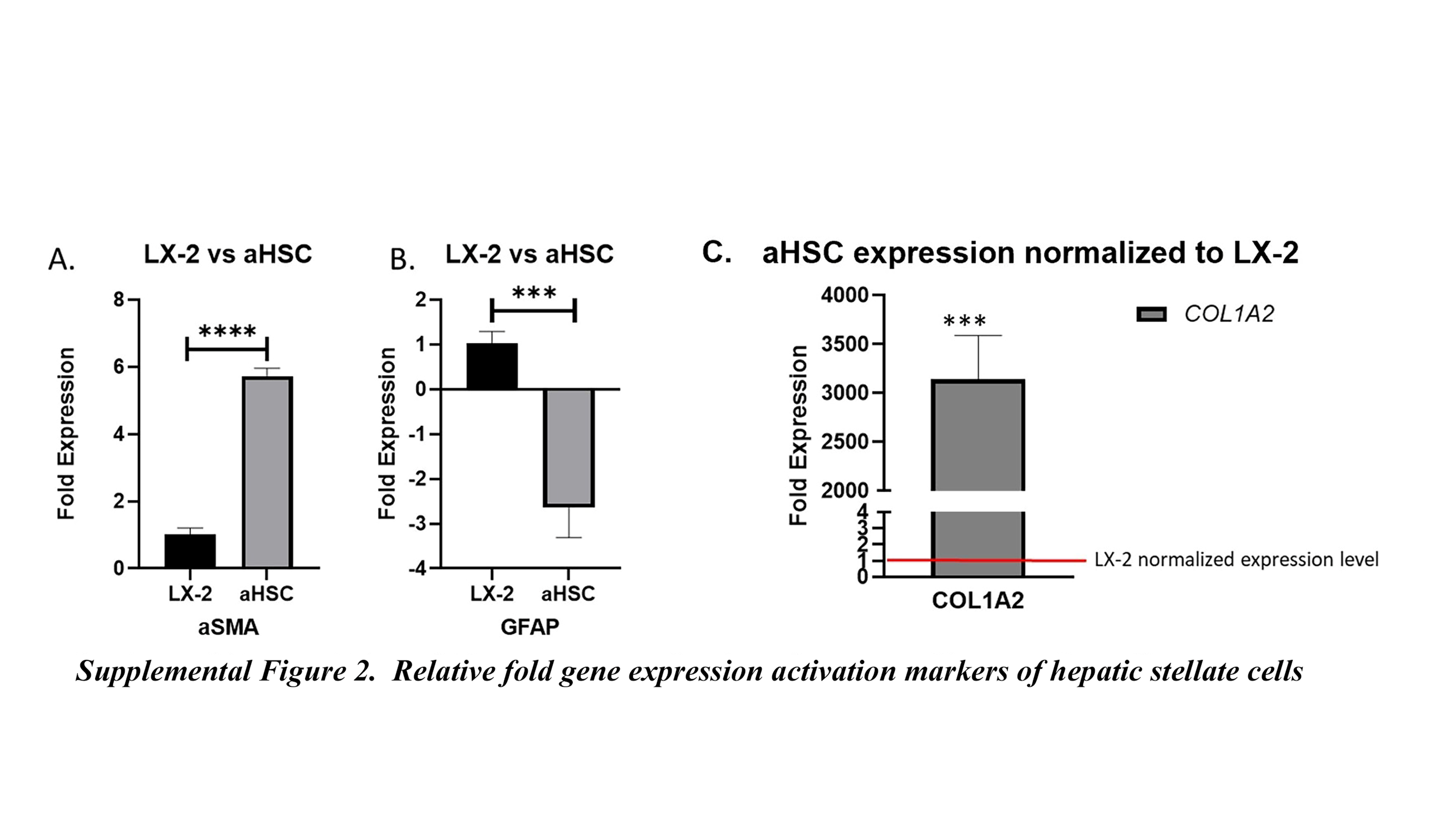

Supplement: Supplementary file 2 — Figure S2 Relative gene expression of activation markers in hepatic stellate cells. A and B, α‐SMA (A) and glial fibrillary acidic protein (GFAP) (B) expression in LX‐2 and aHSC cells. The expression levels were determined by the Livak method, by normalization to the housekeeping gene β2M. C, Collagen IA2 (COL1A2) expression in aHSC cells compared (normalized) with LX‐2 cells. [file BTM2-6-e10207-s002.TIF]

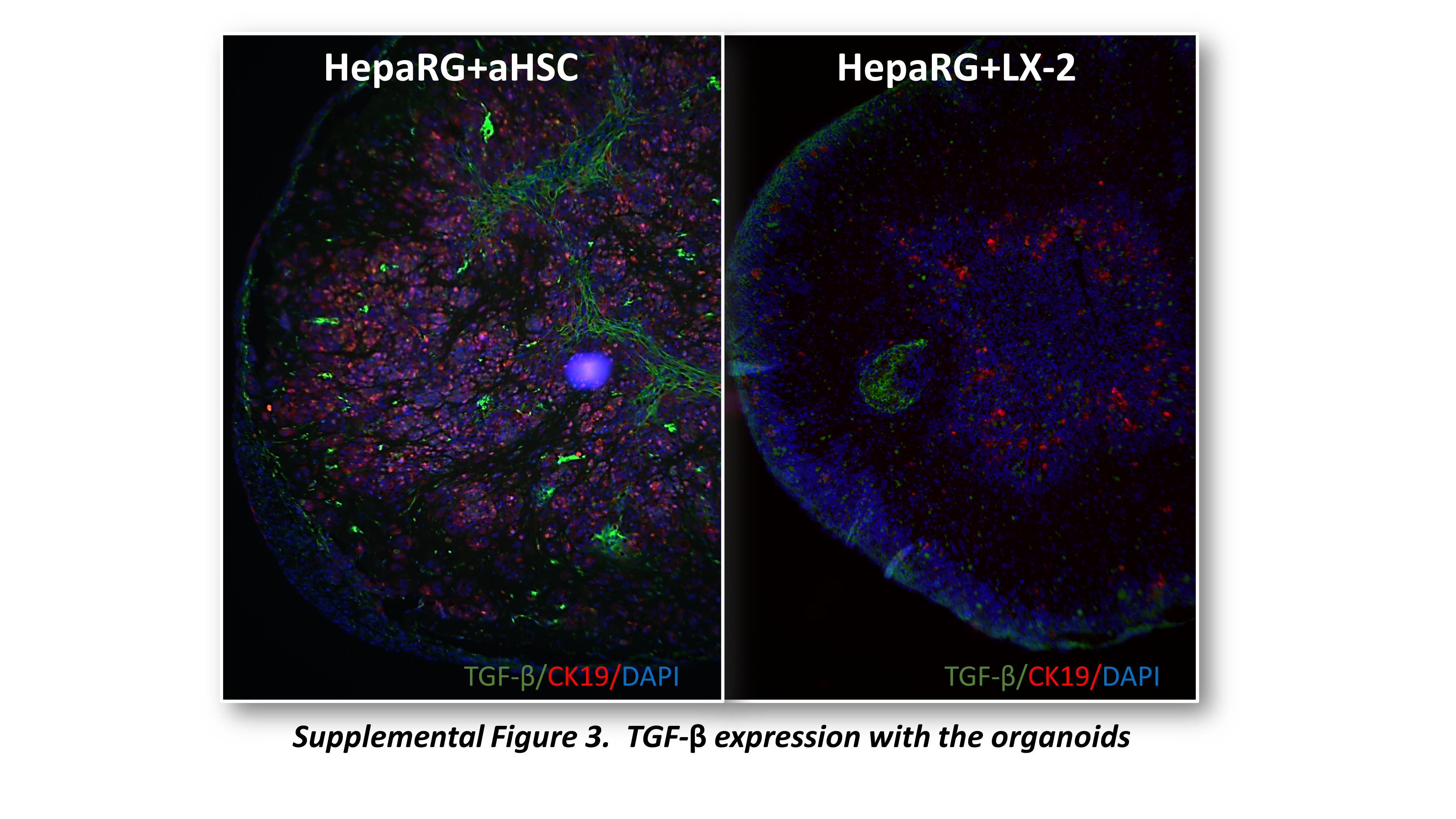

Supplement: Supplementary file 3 — Figure S3 IFC staining of TGF‐B in fibrotic organoids. Immune‐fluorescence staining of TGF‐β in organoids reveals higher TGF‐β expression in HepaRG+aHSC organoids compared with HepaRG+ LX‐2 organoids. Additionally, TGF‐β distribution was observed throughout the HepaRG+aHSC organoid, whereas in HepaRG+LX‐2 organoids it is largely located along the periphery. [file BTM2-6-e10207-s003.TIF]

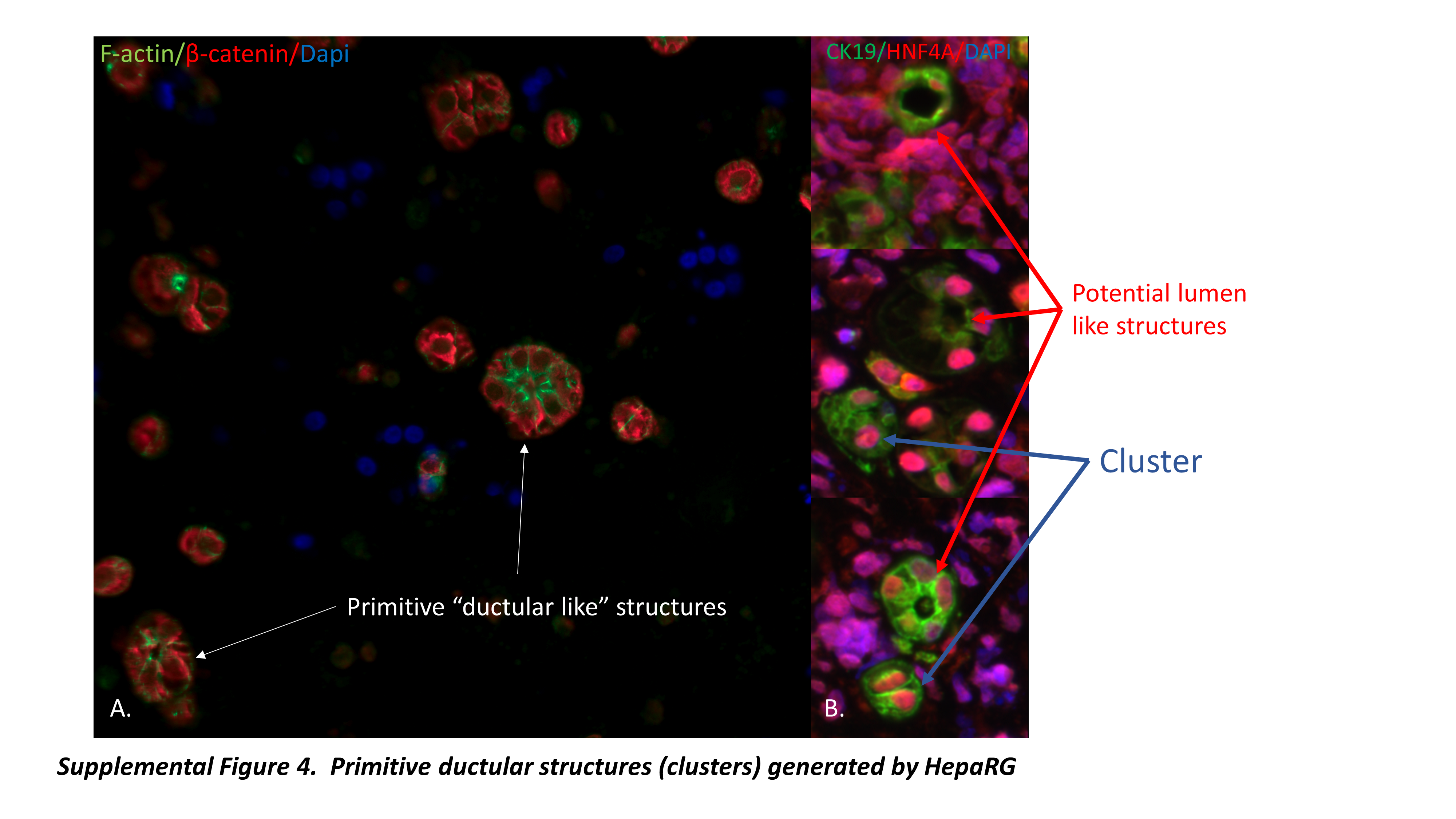

Supplement: Supplementary file 4 — Figure S4 Primitive ductular structures (clusters) generated by HepaRG. A, HepaRG form primitive ductular structures when cultured in 3D constructs. Fully mature biliary ducts express F‐actin in the lumen only and β‐catenin in between. These appear to be immature ducts. B, Cell clusters and lumen‐containing structures observed within the organoids. [file BTM2-6-e10207-s001.TIF]

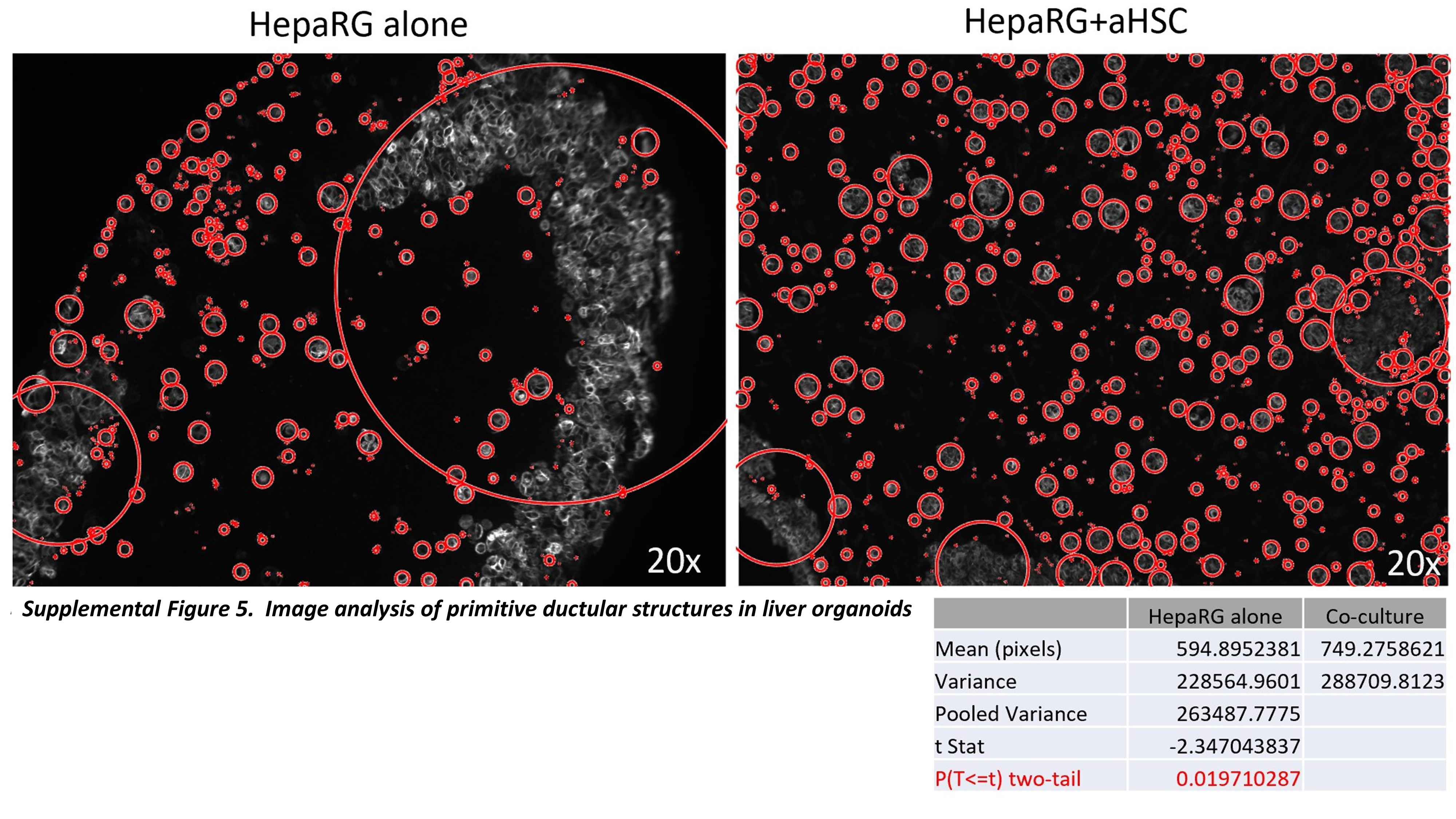

Supplement: Supplementary file 5 — Figure S5 Image analysis of primitive biliary structures in liver organoids. HepaRG and HepaRG+aHSC organoids, as indicated, were harvested for analysis after 1 week in culture. A Matlab script was generated to identify CK19+ duct‐like structure within the image. Regions of interest (ROI's) were thresholded for size, removing large and small outliers. Results show the mean and variance of the number of CK19+ duct‐like structures in HepaRG and HepaRG+aHSC organoids. Organoids containing HepaRG+aHSC cocultures showed higher numbers of CK19+ cell clusters compared with HepaRG alone organoids. [file BTM2-6-e10207-s004.TIF]
